# The roles of mid-myocardial and epicardial cells in T-wave alternans development: a simulation study

**DOI:** 10.1186/s12938-018-0492-6

**Published:** 2018-05-08

**Authors:** D. Janusek, J. Svehlikova, J. Zelinka, W. Weigl, R. Zaczek, G. Opolski, M. Tysler, R. Maniewski

**Affiliations:** 1 Nalecz Institute of Biocybernetics and Biomedical Engineering, Polish Academy of Sciences, 4 Ks Trojdena Str., 02-109 Warsaw, Poland; 2 Institute of Measurement Science, Slovak Academy of Sciences, Bratislava, Slovakia; 3 Department of Surgical Sciences/Anaesthesiology and Intensive Care, Uppsala University, Akademiska Hospital, Uppsala, Sweden; 4 Department of Cardiology, Central Clinical Hospital of Medical University of Warsaw, Warsaw, Poland

**Keywords:** T-wave alternans, Heart model, ECG signal simulation

## Abstract

**Background:**

The occurrence of T-wave alternans in electrocardiographic signals was recently linked to susceptibility to ventricular arrhythmias and sudden cardiac death. Thus, by detecting and comprehending the origins of T-wave alternans, it might be possible to prevent such events.

**Results:**

Here, we simulated T-wave alternans in a computer-generated human heart model by modulating the action potential duration and amplitude during the first part of the repolarization phase. We hypothesized that changes in the intracardiac alternans patterns of action potential properties would differentially influence T-wave alternans measurements at the body surface. Specifically, changes were simulated globally in the whole left and right ventricles to simulate concordant T-wave alternans, and locally in selected regions to simulate discordant and regional discordant, hereinafter referred to as “regional”, T-wave alternans. Body surface potential maps and 12-lead electrocardiographic signals were then computed. In depth discrimination, the influence of epicardial layers on T-wave alternans development was significantly higher than that of mid-myocardial cells. Meanwhile, spatial discrimination revealed that discordant and regional action potential property changes had a higher influence on T-wave alternans amplitude than concordant changes. Notably, varying T-wave alternans sources yielded distinct body surface potential map patterns for T-wave alternans amplitude, which can be used for location of regions within hearts exhibiting impaired repolarization. The highest ability for T-wave alternans detection was achieved in lead V1. Ultimately, we proposed new parameters *Vector Magnitude Alternans* and *Vector Angle Alternans*, with higher ability for T-wave alternans detection when using multi-lead electrocardiographic signals processing than for single leads. Finally, QT alternans was found to be associated with the process of T-wave alternans generation.

**Conclusions:**

The distributions of the body surface T-wave alternans amplitude have been shown to have unique patterns depending on the type of alternans (concordant, discordant or regional) and the location of the disturbance in the heart. The influence of epicardial cells on T-wave alternans development is significantly higher than that of mid-myocardial cells, among which the sub-endocardial layer exerted the highest influence. QT interval alternans is identified as a phenomenon that correlate with T-wave alternans.

## Background

Electrocardiography (ECG) is the most commonly used noninvasive method of assessing heart function, with cardiac arrhythmia representing one of the pathologies that can be detected by this approach. Among cardiac arrhythmias, those of ventricular origin can have serious clinical consequences, such as sudden cardiac death (SCD), which is currently considered the leading cause of cardiovascular mortality in developed countries [1]. The possibility of electrocardiographic risk stratification of such life-threatening ventricular arrhythmias is currently under investigation. In particular, T-wave alternans (TWA) testing is a relatively new method, which is still being developed, for assessing the risk of SCD. TWA is defined as the appearance of periodic beat-to-beat changes in the T-wave amplitude [2], and the prognostic value of such changes has been documented in patients following myocardial infarction [3, 4], chronic heart failure [5], and dilated cardiomyopathy [6]. Accordingly, an understanding of the mechanisms underlying the occurrence of TWA in different diseases is important for the development of antiarrhythmic strategies.

Susceptibility to ventricular arrhythmias, which are associated with the spatiotemporal heterogeneity of the repolarization process in the heart [7, 8], can manifest as the repolarization alternans. This effect, which arises from a beat-to-beat alternation of the action potential (AP) properties in cardiac myocytes, is visible in ECG signals at the body surface as TWA. This process can be spatially concordant if different changes in AP occur in all ventricular cells alternately at even and odd heartbeats. The process can also be discordant if the alternations in at least one region manifest in the opposite phase to that of adjacent myocardium, what would primarily occur when the conduction velocity slows in a spatially limited region or when premature beats arise [9]. Furthermore, the repolarization alternans can induce additional repolarization gradients across the heart wall that are known substrates for cardiac arrhythmias [10]. This process is usually linked to relatively high heart rates (> 110 beats/min). Conversely, at non-elevated heart rates (< 90 beats/min), spontaneous ventricular tachycardia (VT)/ventricular fibrillation (VF) may initiate in patients with systolic dysfunction of the left ventricle [11]. In such instances, TWA is linked to fluctuations in the AP amplitude phase II without AP duration alternans, which is also an indicator of increased vulnerability to VT/VF [12, 13]. TWA can also result from oscillations in the myocyte activation time [9] or in sodium channel inactivation [14]. Notably, our previous studies documented the relationship between TWA and the duration of the refractoriness reflected by the QT alternans [15]. Another factor that influences TWA is anti-arrhythmic pharmacotherapy, which decreases the prevalence of TWA in patients with chronic ventricular tachyarrhythmia receiving amiodarone [16].

However, despite the convincing evidence that TWA is closely associated with the development of re-entrant ventricular arrhythmia and SCD, it is not known whether or how T-wave alternans is linked to the underlying mechanism of ventricular arrhythmia. It is very difficult to model focal arrhythmia triggers as well as map their origin and mechanisms in intact hearts. A limited number of research studies have reported intracardiac alternans during simulation [17, 18] and experimental studies [10, 19, 20]. Specifically, a non-uniform distribution of TWA was observed during the development of discordant alternans, which is a well-known substrate of re-entrant arrhythmia [21]. Previous studies observed a strong correlation between changes in the T-wave amplitude and the QT interval in the ECGs of TWA positive patients [15], although the mechanisms underlying these correlations remains unclear. In comparison, an association with a restitution phenomenon has been suggested, involving time-dependent recovery of potassium channels as a function of the diastolic interval [20, 22–24]. In any case, a deeper understanding of the cardiac pathophysiology related to TWA might shed light on an optimal strategy for preventing the incidence of ventricular arrhythmias and SCD.

Recent progress in computer science has enabled modeling of cardiac electrical fields while also accounting for cardiac anatomy, electrophysiological processes in the cell membrane as well as in the myocardial tissue, and the inhomogeneity of the chest volume [25]. In particular, one group proposed a simplified model of the heart ventricles [26], which was subsequently improved by others [25, 27], that allows for the simulation of heterogeneous ventricular repolarization under normal and pathological conditions [28–30].

In this study, we aimed to simulate the mechanisms responsible for generating TWA in different spatial locations of the heart (including concordant, discordant, and regional discordant, hereinafter referred to as “regional”, origins of TWA), and at different depths (including individual layers of the heart wall), in order to identify the unique relationships between size and location of the region that are responsible for TWA production inside the heart, as well as the TWA amplitude patterns observed at the body surface. In addition, depth analysis would allow for an independent assessment of the influence of subsequent layers of the heart wall on the spatial distribution of the TWA amplitudes on the surface of the body. Moreover, evaluation of the methods that are typically used for TWA detection would enable the development of new parameters for facilitating the identification of patients at risk of ventricular arrhythmias and sudden cardiac death. Lastly, correlation between TWA alternans and QT alternans was studied.

## Methods

### Simulation of ECG signals

**Fig. 1:**
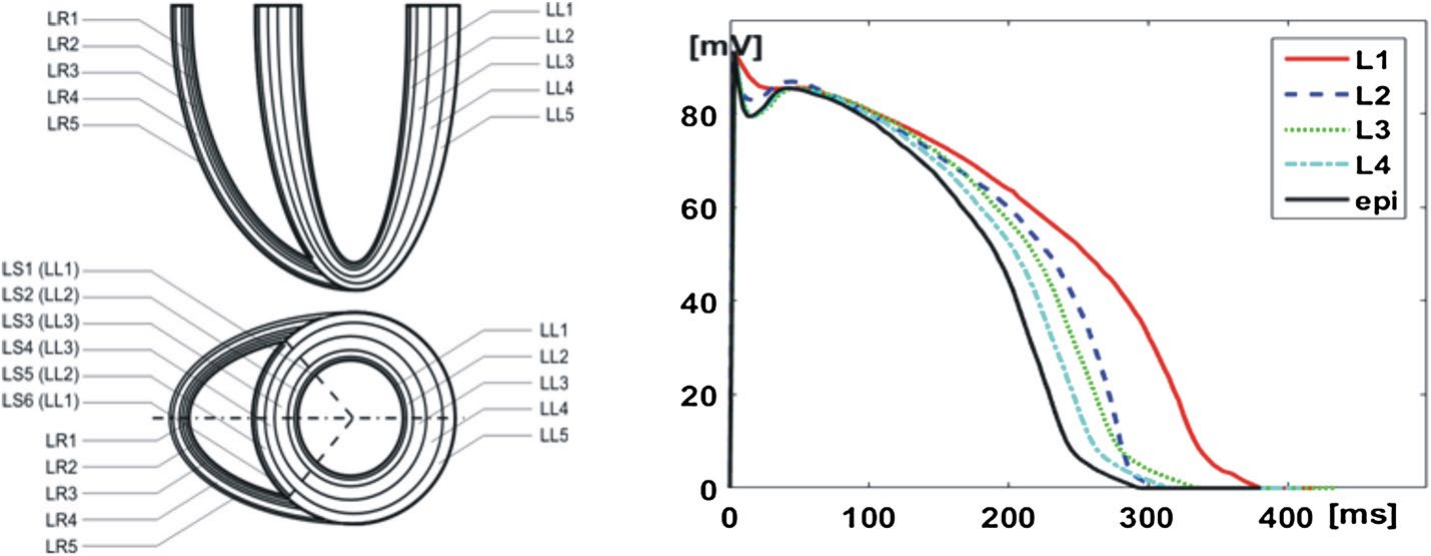
Left: cross-section of the ventricles of the heart model with five layers in each ventricle. Right: action potential curves simulated in mid-myocardial layers (L1, L2, L3, L4), and epi layer (L5)

We utilized the simplified heart model to simulate TWA generation in ECG signals using a solution of the forward problem of electrocardiography. The model of cardiac ventricles was defined by parts of ellipsoids creating five-layered walls in the left and right ventricles, from the inner endocardial layer (L1) through the mid-myocardial layers (L2, L3, L4) to the outer epicardial layer (L5, termed “*epi”* in the study) [26] (Fig. 1). The whole modeled volume was discretized to a 1 mm^3^ mesh grid, and the properties of myocardial cells were assigned to each grid element. For TWA simulation, the actual shapes, amplitudes, and durations of the myocyte APs and the activation propagation velocity were individually predefined for each layer, according to the simulation study reported by Tyšler et al. based on the experimental data measured by Yan et al. in canine left ventricular wedge preparation, to produce corresponding ventricular gradients (Fig. 1) [27, 31]. The parameters of the used heart model were adjusted to obtain the realistic T-wave at the surface of the torso. The AP duration (APD) for the endocardial layer, representing Purkinje fibers was set as 378 ms, while the APD in the other layers varied from 292 to 334 ms. The considerably longer APD in endocardial cells representing Purkinje fibers, as used in our simulations, were reported previously by Yan et al. and Boukens et al. [31, 32].

In the modeled endocardial layer L1, the activation propagation velocity during depolarization was three times faster than that in the remaining parts of the ventricles to simulate the sub-endocardial Purkinje fibers. This parameter was not a subject of the study. The modeled mid-myocardial layers L3 and L4 have the electrophysiological properties different from those of the cells of the endocardium or the epicardium, and are characterized by a prolonged repolarization phase [33, 34]. The modeled AP durations allowed for obtaining realistic simulated ECG signals at the surface of the torso.

The starting points for AP propagation in the simplified heart model were selected according to Durrer’s conclusions [35]. APs later developed into spherical or ellipsoidal wave fronts using cellular automaton principles, and the algorithm allowed for the inclusion of real values of conduction velocity (0.5 m/s [36]) during simulation. At each time point, the actual elementary dipole moment was computed at each point of the grid volume as a difference between the AP at the actual point and that at its closest neighbours in 3D coordinate directions. Thus, the resulting electrical generator of the ventricular myocardium was computed in the form of multiple dipoles at each time point [25]. The inhomogeneous torso model included lung lobes and ventricular cavities, which exhibited conductivities four-times lower and three-times greater than the mean conductivity of the torso, respectively. ECG signals *s(t)* were computed at each time point from the equivalent electrical heart generator at all surface points of the torso model, using the boundary element method according to formula (1) [37]:

1 $$s(t)\, = \,Td(t)$$

where *T* is the time-independent transfer matrix representing the geometrical properties of the torso as an inhomogeneous volume conductor and *d(t)* is the vector of the dipole moments of the equivalent heart generator at each time point.

To study the influence of the measurement scenario on the ability for TWA detection methods, 192-lead body surface potential maps (BSPM), 12-lead ECGs and vectorcardiographic (VCG) signals were simulated.

### TWA modeling

The shape of an electrocardiographic T-wave is the result of voltage gradients within the modeled volume of ventricular myocardium during the repolarization process [27]. These gradients are dependent on the heterogeneity of the duration and shape of the APs relative to their location. For the TWA, two consecutive heartbeats with different AP parameters were simulated to show alternating changes in the repolarization process. We used two models for TWA generation: simulation of periodic changes in AP duration and simulation of periodic changes in AP amplitude phase II. These changes were simulated globally in the whole left and right ventricles to simulate concordant TWA, and locally in selected regions to simulate discordant and regional TWA. Additionally, simulations were carried out separately in each heart wall layer.

**Fig. 2:**
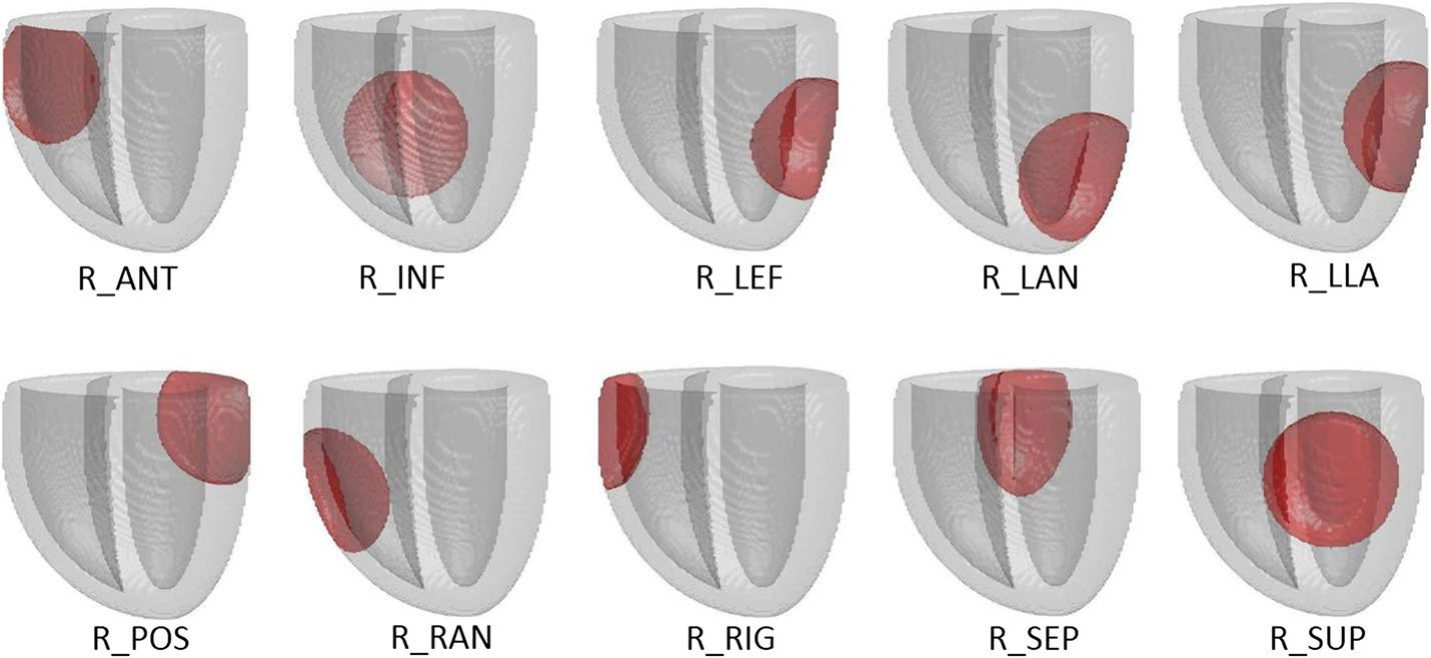
The positions of the modeled regions with changed APD in every even and odd heart beat. The heart model is in vertical position with left ventricle on the right side

The AP duration alternans was simulated by lengthening/shortening of AP duration in two consecutive heartbeats. For the concordant TWA (*CONC*), global AP duration changes were modeled by shortening the AP duration by 4% in the first heartbeat and lengthening it by 4% in the second heartbeat, in relation to the reference simulation [17, 38]. Meanwhile, the discordant TWA simulations *(DISC)* consisted of AP duration changes of 4% in two separate complementary heart regions. If during the first heartbeat the AP duration was shortened in the first region and lengthened in the second region, then in the second heartbeat, the converse changes were made. The following pairs of complementary regions were selected for discordant TWA simulations: the posterior and anterior wall (*D_P/A*), the left ventricle and right ventricle (*D_L/R*), and the apex and base (*D_A/B*). Regional TWA was modeled as a 10% change in AP duration at selected regions of the myocardium. The following locations described by the American Heart Association standard nomenclature were used analogically in both ventricles: right basal anterolateral—anterior (*R_ANT*), left mid inferior—inferior (*R_INF*), left mid anterolateral—left (*R_LEF*), left apical anterior—left anterior (*R_LAN*), left mid lateral—left lateral (*R_LLA*), left basal inferolateral—posterior (*R_POS*), right basal lateral—right (*R_RIG*), right mid anterolateral—right anterior (*R_RAN*), left mid anterior—superior (*R_SUP*), and right basal inferoseptal—septum (*R_SEP*) as shown in Fig. 2.

The AP amplitude alternans was simulated by modifying the AP amplitude, via a 10% decrease in the amplitude, during the repolarization phase (phase II) in a second simulated heartbeat. All simulations were carried out separately at mid-myocardial layers (L2, L3, L4 layers), and *epi* layer (L5 layer). A different proportion of the modeled ventricular volume resulting mainly from the different thickness of the modeled left and right ventricular walls was utilized for each type of TWA simulation, as described in Table 1.

**Table 1:** Volumes of the heart (the percentages of the whole heart model = 100%) wherein AP parameters were subject to changes for TWA simulation

TWA type	Mid-myocardial layer percentage of heart vol. (%)		Epicardial layer percentage of heart vol. (%)	
CONC	62.19		25.09	
D_L/R	Left: 48.23	Right: 13.96	Left: 16.68	Right: 8.41
D_A/B	Apex: 27.85	Base: 34.34	Apex: 13.28	Base: 11.81
D_P/A	Post: 32.08	Anter: 30.11	Post: 12.76	Anter: 12.33
Regional	2.40–8.53		0.50–3.57	

*CONC* concordant, *D_L/R* discordant left and right, *D_A/B* discordant apex and base, *D_P/A* discordant posterior and anterior

### TWA actual recordings

To compare the results obtained by simulation with actual data, recordings were acquired from two TWA-positive patients with implantable cardioverter defibrillator (ICD). The medical characteristics of the patients are presented in Table 2.

**Table 2:** Medical data of TWA positive patients

	Patient 1	Patient 2
Sex	Man	Woman
Age	63 years old	68 years old
Underlying disease	CAD, 2 × MI	CAD, 1 × MI
LVEF (%)	68	30
Implantation type	ICD (VVI)	ICD (VVI)

*LVEF* left ventricle ejection fraction, *CAD* coronary artery disease, *MI* myocardial infarction, *ICD* implantable cardioverter defibrillator, *VVI* type of heart pacing: the ventricles are paced when the intrinsic ventricular rhythm falls below the pacemaker threshold

Specifically, we obtained 2-min ECG recordings during the ventricular electrical pacing at 100 bpm, with the use of ICD electrodes. Stimulation was applied to increase the heart rhythm, which in turn increases the TWA amplitude, and TWA calculations were carried out using the differential method [39]. This study was performed in accordance with the principles set forth in The Declaration of Helsinki and was approved by the Ethics Committee of the Medical University of Warsaw, Poland (KB/167/2006). All participants provided informed consent prior to their participation.

### TWA parameters

For quantitative assessment of the TWA, a mean T-wave (*T*_*mean*_) value was computed for each simulated signal as the sum of the signals forming the T-wave divided by the number of samples in the T-wave time interval, according to formula (2):

2 $$T_{mean} = \mathop \sum \limits_{{i = t_{start} }}^{{t_{end} }} \frac{{s_{i} }}{{t_{end} - t_{start} }}$$

where *s*_*i*_ was i-th sample of the ECG signal, *t*_*start*_ was selected as a J-point at the ECG signal, and *t*_*end*_ was selected as the time instant after maximal T wave amplitude when the root mean square value of the ECG signal was less than or equal to 0.005 mV.

The T-wave mean was selected for TWA calculations to be independent from the location of the TWA maximum within T-wave, and for the reduction of noise. Then, mean vectors (*T*_*VCG/12leads/BSPM*_) consisting of the mean values for each simulated T-wave were defined with the dimension dependent upon the considered measuring system: [1 × 3] for VCG, [1 × 12] for 12-lead ECG, and [1 × 192] for BSPM. For the corresponding consecutive heartbeats producing even and odd mean vectors, the parameters *Vector Magnitude Alternans* (*VMA*) and *Vector Angle Alternans* (*VAA*) were computed using formulas (3) and (4), respectively:

3 $$VMA_{VCG/12leads/BSPM} \, = \,\frac{{\left\| {\overrightarrow {{T_{VCG/12leads/BSPM\_even} }} } \right.\, - \,\left. {\overrightarrow {{T_{VCG/12leads/BSPM\_odd} }} } \right\|}}{N}$$

4 $$VAA_{VCG/12leads/BSPM} = arccos\frac{{\overrightarrow {{T_{VCG/12leads/BSPM\_even} }} \overrightarrow {{T_{VCG/12leads/BSPM\_odd} }} }}{{\left\| {T_{VCG/12leads/BSPM\_even} } \right\| \,\left\| {T_{VCG/12leads/BSPM\_odd} } \right\|}}$$

where $\left\| T \right\|$ represents the Euclidean norm of vector *T* and *N* represents the number of leads used for measurement.

Considering each measured signal separately (*T*_*even*_ and *T*_*odd*_), the TWA amplitude was calculated using the Differential Method [39] as a difference between the mean values of two consecutive T-waves (*T*_*even*_ and *T*_*odd*_) according to formula (5):

5 $$TWA_{VCG/12leads/BSPM,n} = \left| {T_{even,n} } \right. - \left. {T_{odd,n} } \right|$$

where vertical bars indicate absolute values.

The ability for TWA detection in a standard 12-lead ECG system was studied by identifying the lead with the most frequent occurrence of the maximum TWA amplitude value.

The BSPMs of TWA amplitude were used to observe the spatial distribution of the TWA amplitude on the surface of the torso. Owing to the large diversity of the simulated TWA amplitudes, the scale for each drawing was set individually. Both noise and interferences were not included, which enabled the acquisition of the distributions of TWA amplitudes on the body surface, including those below the detection threshold for most of the generally used detection methods when analyzing real data (10 μV) [40]. In this way, all patterns of TWA amplitude distribution on the torso could be analyzed. The outcome of this study will be of particular interest when researching real signals from patients with stronger TWA generators than those simulated in this work.

The QT alternans phenomenon was also studied to support previous experimental findings that showed a strong correlation between the QT interval and the T-wave amplitude in TWA-positive patients [15]. The QT interval was calculated as the time interval between the Q point and *T*_*end*_, and the QT alternans was computed as the difference between QT intervals calculated during two consecutive heartbeats from the 12-lead ECG.

## Results

### TWA simulation by AP duration alternans

#### Concordant TWA

**Fig. 3:**
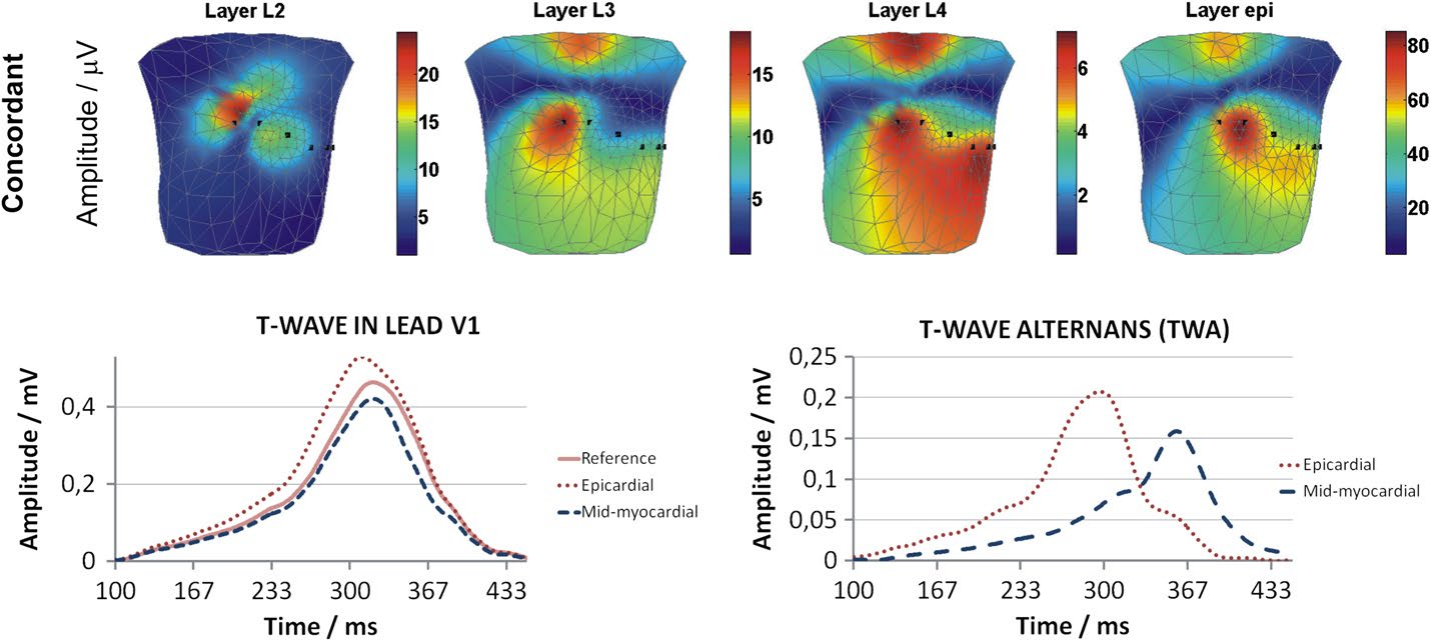
Body surface distributions of the concordant TWA amplitudes simulated by AP duration alternans for heart model mid-myocardial layers (L2, L3, L4 layers), and epi layer (upper); The T-wave signals in lead V1 simulating concordant TWA in mid-myocardial and epi (epicardial) layers (lower left); Concordant TWA simulated by AP duration alternans in mid-myocardial and epi (epicardial) layers (lower right)

The concordant TWA simulated by shortening/lengthening the AP duration, relative to the reference signal (solid line), in the mid-myocardial and *epi* layers in lead V1 is shown in Fig. 3 (lower). The BSPMs of the TWA amplitude are shown in Fig. 3 (upper).

All BSPMs of the TWA amplitudes represent the influence of concordant AP duration alternans simulated in the heart on the surface electrocardiographic potentials. Influences of *epi* and mid-myocardial layers are shown separately. The highest amplitudes of TWA were generated by the AP duration alternans simulated in the *epi* layer, compared to that in the mid-myocardial layers. The BSPM patterns of the TWA amplitudes for the L3, L4, and *epi* layers were similar, with the maximal values, which were split into two regions, being at the same position in each map. Indeed, because of the similarity of these three maps, it was difficult to differentiate which layer was involved in TWA generation. In contrast, the BSPM patterns of the TWA amplitudes originating from layer L2 produced a single maximum, which was shifted toward the upper right side of the body, making this source of disturbances distinguishable from the other layers.

The concordant AP duration alternans in the *epi* layer accelerated the repolarization process, as shown by an earlier T-wave slope occurrence and a T-wave maximum shift toward earlier times in comparison to reference curve (Fig. 2 lower left). Conversely, no shift in T-wave maximum was observed when the concordant AP duration alternans was simulated in mid-myocardial layers.

#### Discordant TWA

**Fig. 4:**
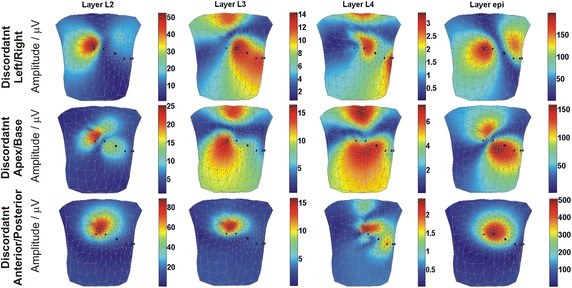
Body surface distribution of the discordant TWA amplitudes upon AP duration alternans simulation in mid-myocardial layers (L2, L3, L4 layers), and epi layer

The distributions of the discordant TWA amplitude simulated by the AP duration alternans are shown in Fig. 4. The change in AP duration was simulated in the mid-myocardial, and *epi* layers, respectively, in three pairs of regions: the anterior and posterior wall, the left ventricle and right ventricle, and the apex and base.

Similar patterns of BSPMs of the TWA amplitudes were observed for all layers of discordant anterior/posterior TWA, as well as in layer 2 of the left/right and apex/base discordant alternans. As a result, the location of the TWA generator could not be distinguished based on the BSPMs of TWA amplitudes in the above-mentioned locations. In contrast, all other body surface TWA amplitude maps differed significantly, and were therefore distinguishable.

#### Regional TWA

**Fig. 5:**
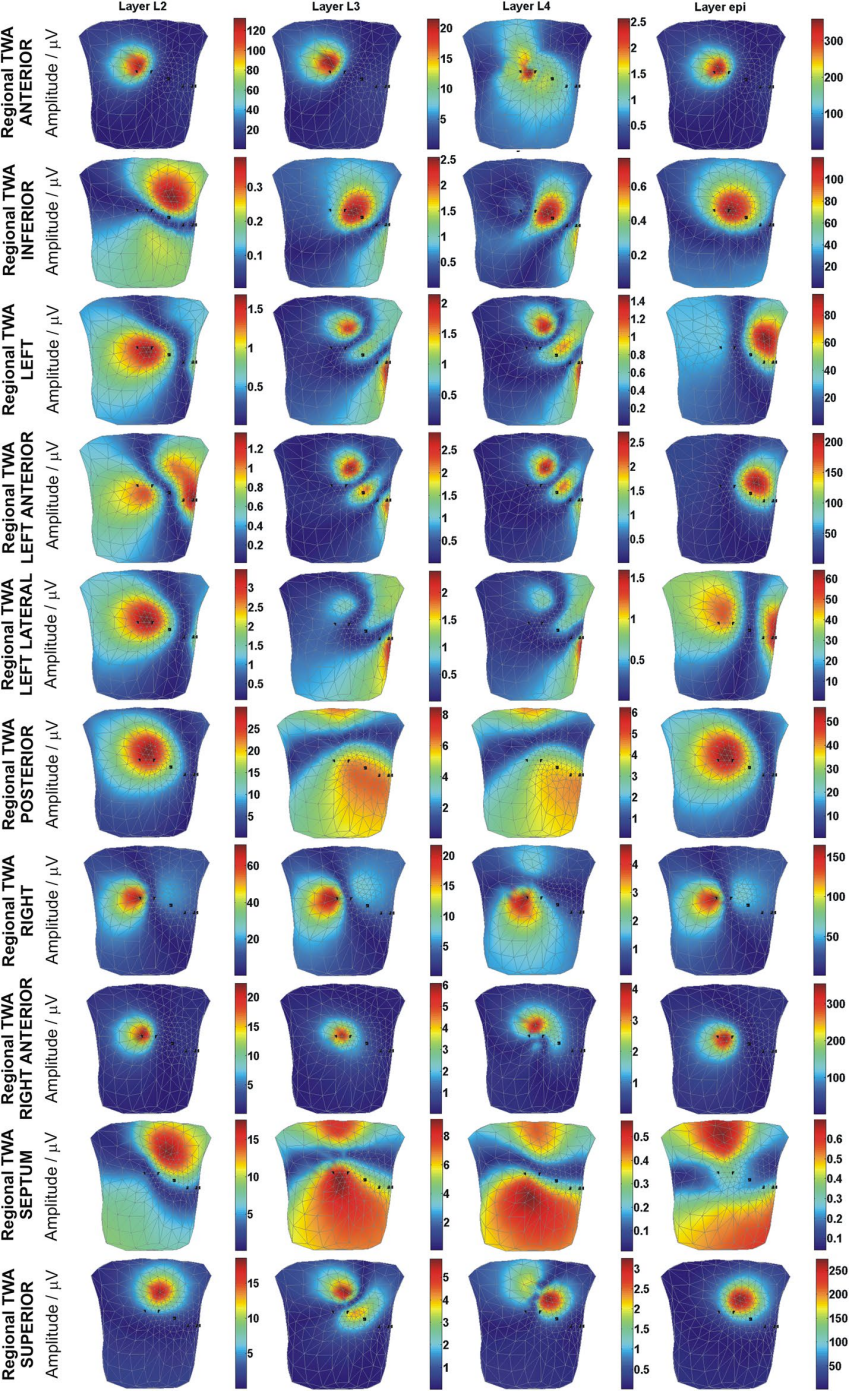
Body surface distribution of the regional TWA amplitudes upon AP duration alternans simulation in mid-myocardial layers (L2, L3, L4 layers), and epi layer

The maps of TWA amplitudes simulated by the AP duration alternans are shown in Fig. 5. The change in AP duration was simulated in the mid-myocardial, and *epi* layers, respectively, in anterior, inferior, left, left anterior, left lateral, posterior, right, right anterior, superior, and septum regions.

The BSPMs of the TWA amplitudes simulated for each region of the heart exhibited distinct patterns between layers, with the exception of those produced by AP duration alternans located in layers L3 and L4 (mid-myocardial) (Fig. 5). These latter findings indicate that depth recognition between layers L3 and L4 will be impossible when comparing maps. Moreover, the patterns of the maps differed significantly for different types of regional TWA. The lone exceptions were in those of the anterior, right anterior, and right regional TWAs, which exhibited similar patterns for all layers. The magnitude of the TWA amplitude also differed between maps. Because of the limited ability for TWA detection of the methods used in clinical practice, some of the computed amplitudes would not be detectable when analyzing real data. Assuming a detection threshold of 10 μV, as suggested previously [40], TWA would be detected when the AP duration alternans was present in L2 layer for the anterior, posterior, right, right anterior, septum, and superior regional TWAs. Meanwhile, for layers L3 and L4, TWA would be detected for only the anterior and right regional TWAs. Conversely, the *epi* layer did not produce detectable amplitudes when the septum regional TWA was simulated. Notably, the above detection analysis would only be accurate when the ECG electrode is located close to the maximum of the TWA amplitude located at the body surface.

### TWA modeling by AP amplitude alternans

#### Concordant TWA

**Fig. 6:**
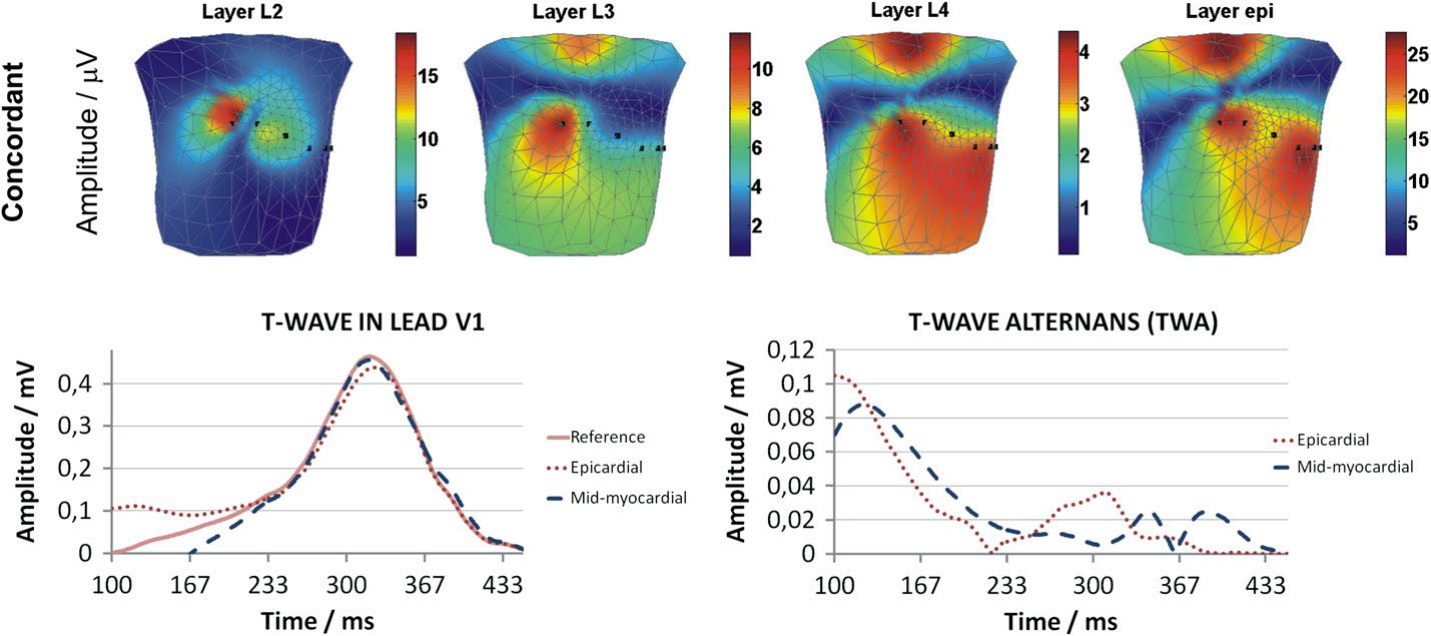
Body surface distributions of the concordant TWA amplitudes simulated by AP amplitude alternans for heart model mid-myocardial layers (L2, L3, L4 layers), and epi layer (upper); The T-wave signals in lead V1 simulating concordant TWA in mid-myocardial and epi (epicardial) layers (lower left); Concordant TWA simulated by AP amplitude alternans in mid-myocardial and epi (epicardial) layers (lower right)

The BSPMs of the concordant TWA amplitudes, as simulated by a 10% decrease of the AP amplitude in phase II, are shown in Fig. 6. The electrocardiographic representation of the concordant TWA in lead V1 was simulated relative to the reference signal (solid line).

The patterns of the body surface TWA amplitudes simulated by AP amplitude alternans are similar to those obtained during simulation of concordant TWA by AP duration alternans. The AP amplitude alternans generated the highest TWA amplitude when simulated in the *epi* layer, compared with that observed in the mid-myocardial layers (L2, L3, and L4 layers); however, the differences between this value for the *epi* and the mid-myocardial layers were smaller than those observed during the AP duration alternans simulations. The body surface TWA amplitude distributions were similar for the L3, L4, and *epi* layers, showing the same positioning of the maximal values, which were split into two regions. However, it would be difficult to distinguish the depth of location of the alternans generator in the heart walls via analysis of the patterns observed in these maps. In contrast to the other simulations, alternans in layer L2 produced a single maximum, which was shifted toward the upper right side of the body. In all simulated maps the maximal amplitude of TWA was above the threshold for detection in real ECG recordings (10 μV). Also, in contrast to the results obtained during AP duration alternans simulations, the ECG representation of alternans showed a dominant TWA amplitude in the early repolarization phase (ST segment elevation), which is usually considered a sign of ischemia and could cause incorrect medical diagnosis. Lastly, the AP amplitude alternans did not significantly change the time point at which the T-wave maximum occurred.

#### Discordant TWA

**Fig. 7:**
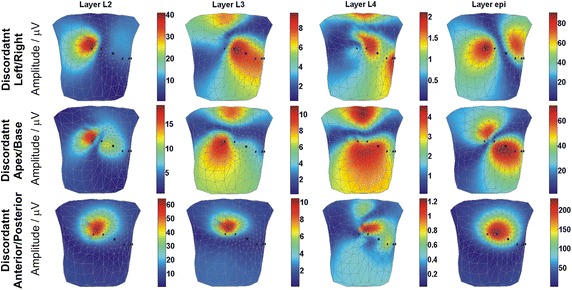
Body surface distribution of the discordant TWA amplitudes upon AP amplitude alternans simulation in mid-myocardial layers (L2, L3, L4 layers), and epi layer

The BSPMs of discordant TWA amplitudes simulated by changes in the AP amplitude in phase II are shown in Fig. 7. The AP duration time was fixed during these simulations.

The body surface TWA amplitude patterns were similar to those obtained during discordant AP duration alternans simulation, with the difference that the TWA amplitudes were lower. The most prominent amplitudes of TWA were observed upon simulation of the anterior/posterior discordant TWA. For anterior/posterior discordant TWA, the body surface TWA amplitude patterns were similar to those obtained during discordant AP duration alternans simulation, with the same positioning of the maximum. Indeed, due to the similarity in the maps obtained from all four layers, it was not be possible to deduce the depth location of the alternans generator. When generating the apex/base and left/right discordant alternans, the TWA amplitude patterns were similar to each other but varied when AP amplitude alternans was simulated in the different layers. Because of that, differentiation between these two types of discordant alternans might be difficult.

### TWA actual recordings

**Fig. 8:**
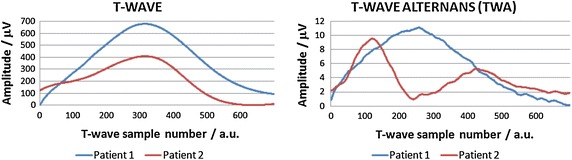
TWA amplitude distribution patterns measured in two patients with implantable cardioverter-defibrillators upon increase of the pacing rate to 100 bpm through electrical stimulation

Patients with ICD and increased vulnerability to ventricular arrhythmia were studied to assess compliance and evaluate the accuracy of the simulated data. Notably, the TWA can affect the shape of the T-wave in different ways. Figure 8 shows the T-wave signals and the TWA amplitudes observed in two patients. The recordings were made using the orthogonal XYZ lead configuration electrocardiographic system, and only leads yielding distinguishable TWA are presented (lead X for Patient 1 and lead Z for Patient 2).

For the Patient 1, the maximal TWA amplitude occurred near the top of the T-wave. This pattern is similar to that observed during our AP duration alternans simulation. Conversely, in the Patient 2, the maximal TWA amplitude occurred at the beginning of the repolarization phase (in the ST segment), followed by smaller changes at the end of repolarization, which was similar to the results obtained during AP amplitude alternans simulation. Moreover, the ranges of amplitudes of TWA observed in these patients corresponded to those obtained during simulations.

### TWA parameters analysis

**Fig. 9:**
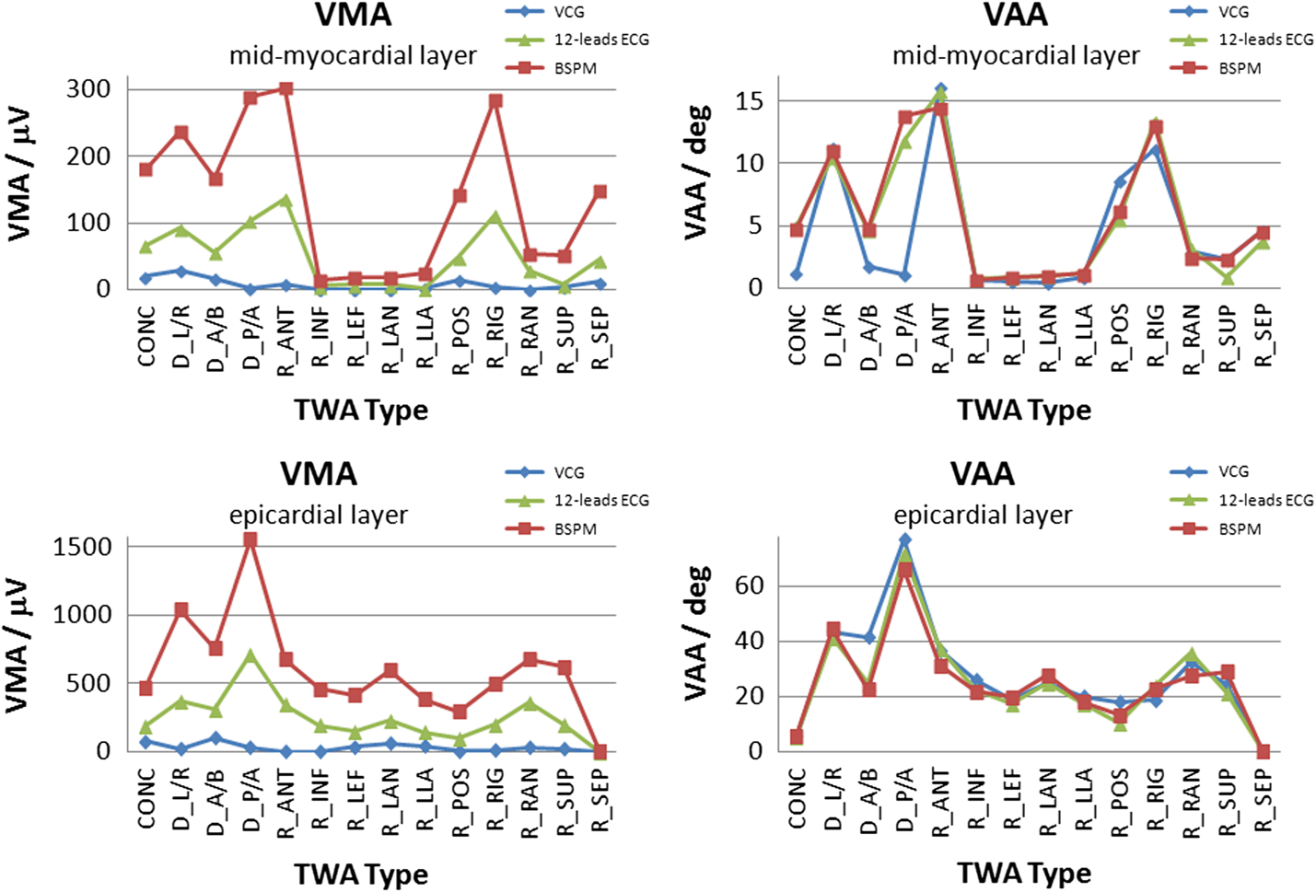
Vector magnitude alternans (VMA) and vector angle alternans (VAA) calculated from BSPM, and 12-lead ECG in the groups wherein the source of TWA was located in the mid-myocardial layers (L2, L3, and L4) or in the epi layer

Mean vectors were computed from VCG, 12-lead ECG and BSPM, and then used for *VMA* and *VAA* calculations. Because it is assumed that small periodical T-wave amplitude variations originate from mid-myocardial regions [24], we focused on the influences of changes in mid-myocardial layers on ECG signals at the torso. Calculations were carried out for each of the types of TWA simulated by changes in AP duration. The results of calculations using *VMA* and *VAA* parameters for concordant, discordant, and regional TWA are presented in Fig. 9. Results obtained for the group in which the mid-myocardial layers were a source of TWA and for the group in which the *epi* layers developed TWA are presented separately.

Notably, while there was a strong correlation between the *VMA* parameter and the number of simultaneously considered leads on the torso, the *VAA* parameter was almost independent of this factor. The highest ability for TWA detection of the *VMA* parameter was reached when the BSPM lead system was applied.

**Fig. 10:**
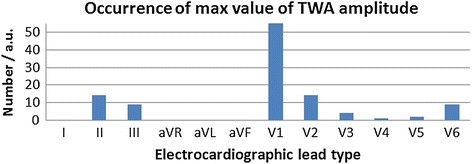
Number of occurrences of the maximum TWA amplitude value in the 12-lead ECG system for 108 simulations

The number of occurrences of the maximum TWA amplitude obtained from the 12-lead ECG was calculated, and is depicted in Fig. 10. Each of the types of TWA simulated, as well as all four layers, was taken into account. In most instances, the maximal TWA amplitude value was obtained from the V1 lead.

**Fig. 11:**
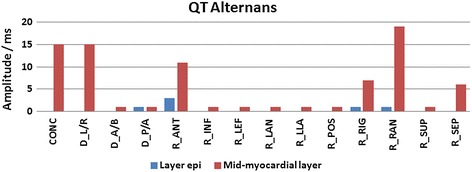
QT alternans as a phenomenon associated with TWA development (periodic QT interval change)

The QT alternans values calculated upon AP duration alternans are separately presented for concordant, discordant, and regional TWA, as shown in Fig. 11. The calculations were carried out separately for the mid-myocardial layers and *epi* layers. Production of QT alternans in surface ECG was carried out via the same process used for generation of TWA.

The QT alternans was primarily influenced by the mid-myocardial layers. Moreover, we detected strong correlations between the presence of QT alternans and the occurrence of concordant, discordant left/right and regional anterior, right, right anterior, and superior TWA.

## Discussion

In the present study, a computerized heart model allowing for the simulation of transmural AP duration and shape heterogeneity was used to investigate putative differences in the source of mid-myocardial and epicardial TWA. The applied model was designed based on the electrophysiological activity of the ventricles and a solution of the forward problem for the ventricular repolarization process. Realistic shapes of standard ECG signals were computed on the body surface, and BSPM representations were used to illustrate the spatial distribution of TWA. These patterns can be applied to localization of the source of TWA within the heart.

### TWA modeling study

The greatest influence on TWA development involved changes in the epicardial layer, which could be most clearly observed via electrocardiographic representation. This can be explained by the fact that the changes of the epicardial and endocardial APD influence the dispersion of repolarization and, consequently, the T wave amplitude. It does not apply to the changes of the midmyocardial APD which does not affect the general magnitude of the dispersion of repolarization. In contrast, among the mid-myocardial layers, changes in the layer closest to the endocardium (L2) were best reflected on the thorax; however, this influence remained weaker than that arising from the epicardium. We hypothesize that this phenomenon may be due to the large difference in AP duration observed between endocardial layers L1 and L2 (Fig. 1). High susceptibility of the L2 layer to AP duration changes may have clinical impact because this layer is exposed to damage such as in the case of hypertension, wherein the sub-endocardial cells are primary affected by permanent higher blood pressure [41]. Notably, changes in location of the alternans generator inside the heart resulted in significant alteration of the BSPMs of the TWA amplitudes (Fig. 5); this effect may allow for the use of such maps as classifiers for identifying the location of the TWA generator within the heart.

In the assessment of SCD risk, the type of TWA is critical [10, 42]. In the current study, discordant and regional changes in AP duration and amplitude yielded greater influences on TWA development than concordant changes. These types of disturbances are directly linked to the mechanism of arrhythmogenesis [10] and may predispose patients to conduction block, a well-known source of reentrant arrhythmia.

During our simulation studies, the location of the region with modified AP was known precisely. Solving the forward problem allows us to obtain the spatial distribution of TWA amplitude on the thorax. We considered that the atlas of patterns of TWA amplitude distributions might be useful in clinical practice for assessing the heart region responsible for TWA generation. This could be performed by matching the pattern obtained from actual electrocardiographic signals (from the patient) with a pattern from the atlas, thus potentially providing information regarding the type of TWA (concordant, discordant, or regional) exhibited by a given patient, as well as the location of the TWA generator.

### TWA in actual data

In the electrocardiographic recordings taken from patients, the most frequently occurring TWA pattern was typically observed close to the maximum of the T-wave amplitude. This type of TWA distribution was confirmed by the results of our simulations upon modification of AP duration. In some patients, however, the maximum of the TWA distribution is located in the QT interval. Notably, similar results were obtained in the current study when both the concordant and discordant TWA were simulated via alteration of the AP amplitude in phase II. Thus, our results are consistent with those obtained by other groups [12, 13] indicating that changes in AP amplitude might also be responsible for TWA generation. Moreover, the amplitudes of the simulated TWA (in the range of tens of microvolts) were similar to those observed in our in vivo measurements, confirming that our computed model could accurately simulate the pathologies assessed in this study.

### TWA parameters

In the current study, two parameters that could be of importance for TWA assessment and stratification of the risk of ventricular arrhythmia occurrence were proposed. The ability for TWA detection of these parameters was studied for different types and locations of AP disturbance in the heart model. Among the results obtained for mid-myocardial changes, the highest values of *VMA* and *VAA* were reached for the regional, anterior TWA simulation. The values of both newly introduced parameters (*VMA* and *VAA*) were approximately five times greater for changes in the epicardial layer than for the changes in mid-myocardial layers. Furthermore, *VAA* was almost independent from the number of simultaneously considered leads on the torso. In comparison, *VMA* increased distinctly with a higher number of leads. Additionally, the occurrence of relatively small *VMA* values for mid-myocardial layers may correspond with the hypothesis that mid-myocardial regions are responsible for slight but periodic changes in T-wave amplitude [43]. Finally, the changes responsible for the TWA generation present in the epicardium may overshadow all the other inner sources, and is likely easily visible within ECG signals.

The highest *VMA* values were obtained when BSPM signals and their TWA amplitudes were taken into account. The ability for TWA detection of this parameter was even higher than the TWA calculated directly from lead V1, which represents the ECG lead wherein the maximum value of the TWA amplitude occurred most often (Fig. 10) [17]. Therefore, we recommend using the *VMA* parameter in TWA detection. However, there were several regions in the left ventricle where none of the parameters reached the diagnostically significant value of 10 μV when BSPM was used (Fig. 9). We therefore could assume that TWA could not be detected in the following parts of the heart: inferior (*R_INF*), left (*R_LEF*), left anterior (*R_LAN*), left lateral (*R_LLA*), and superior (*R_SUP*). However, confirmation of these assumptions may be very difficult in the actual organ.

QT alternans, which is generated by the same processes that are responsible for TWA development, appears to be an important phenomenon in TWA detection. As shown in Fig. 11, QT alternans may be generated by AP duration alternans located in the mid-myocardial, but not *epi*, layers of the heart. To the best of our knowledge, this is the first indication of such origins of the QT alternans generator. Furthermore, QT alternans appearance was strongly connected with the type and spatial location of the repolarization alternans in the heart. The period of myocardial depolarization is terminated by repolarization process which is linked with downstroke of the action potential and the T-wave in the electrocardiogram. Because APD determines QT duration, any changes in APD, in particular periodic, are predisposed to reflect on QT interval duration. However, the impact of the APD changes depends on the position of the corresponding cells within the myocardium where they were manifested. APD affect on the amplitude parameters of the cardiac electric field as it can be seen by comparing Fig. 9 (VMA for midmyocardial cells) and Fig. 11. QT alternans correlate with the VMA in most of the cases, except of the case D_A/B (where VMA is large and QTA is small) and R_RAN (where in contrary VMA is small and QTA is large). The mentioned exceptions confirm that the influence of APD changes on electrocardiographic signals is location dependent. Specifically, this effect only developed when concordant, discordant left/right, regional anterior, right, right anterior, and septum were simulated. Notably, coexistence of QT alternans could influence TWA measurements. Many of the detection procedures used in the preprocessing stage utilized a constant QT time interval for T-wave detection and extraction from ECG signals. It is further used for TWA calculation. Additionally, QT alternans could influence T-wave location according to the window of analysis with beat-to-beat frequency. Depending on the TWA detection method, it may produce a significant TWA value at the output of the detector. In this way, QT alternans may mimic increases or decreases in real TWA amplitudes, thereby influencing the positive predictive value of alternans testing. Appearance of QT alternans during TWA simulations might be associated with the restitution phenomenon, which depends on changes in AP duration during systolic and diastolic periods [10].

The limitation of the current study comprises the simplified geometry and simulation scenario of AP propagation, with predefined AP shapes and transmural AP duration heterogeneity as the only baseline APD gradient. The recent studies such as done by Opthof et al. and Boukens et al. indicated, that in in vivo experiments the transmural gradient is not normally predominant [44, 45]. In the work done by Arteyeva et al. it was concluded that the T-wave genesis depends on an interaction of several gradients (apex-base, anterior–posterior, RV–LV) [46]. However, the presence of the transmural APD gradient is crucial for genesis of the T-wave [47]. In the current study, the other mentioned gradients were simulated as possible generators of TWA. While the steady APD heterogeneity is responsible for the amplitude of the T-wave itself, TWA observed in body surface ECG’s is the result of periodical changes of APD of the myocardial cells that reflect as alternating T-wave amplitude in consecutive heart beats. The main goal of the present simulation work was to study the influence of the APD heterogeneity disturbances on the T-wave amplitude in simulated ECG signals at the surface of the torso. In spite of its simplicity the proposed heart model provide valuable insights regarding the influences of changes of myocardial cells properties on the ECGs at the surface of the torso. The proposed heart model has also been utilized in other studies to help interpret the ECGs of patients with myocardial ischemia and infarction. The results were consistent with ECG signals acquired from patients [28–30]. In the more advanced models such as monodomain or bidomain the shape of the APs and their propagation are computed simultaneously and the electrotonic coupling is assumed [48]. The influence of intramyocardial changes on TWA generation could be later studied on more detailed heart models.

The influence of the fiber anisotropy of the ventricular muscles on ECG signals resulting in different activation propagation velocity along and radially the muscle fibers as it was shown in the study of Streeter et al. [49] and later developed in other works [50, 51] was omitted in presented work. The fiber anisotropy is the steady property of the myocardium and thus it could be neglected when the effect of the periodical changes of APD is studied.

The present simulation study used two possible transient mechanisms of TWA development: local changes in AP duration or AP amplitude at the cellular level. Deeper investigation into the effects of both types of transient changes at the molecular dynamic level, for example in myocytes, as conducted by Rudy et al. [52], may provide more precise information regarding the mechanisms of TWA generation.

## Conclusion

In this study, various types of concordant, discordant, and regional TWA were simulated by altering the AP duration or amplitude of myocytes. The effects of such changes were stronger when carried out in the epicardial layer than in the mid-myocardial layers. These findings support the hypothesis promoting the mid-myocardial responsibility for TWA development in actual signals with very small amplitudes, which are barely detectable in clinical observation.

We proposed two parameters, *VMA* and *VAA*, as possible indicators of TWA. The highest *VMA* values were observed in body surface maps, supporting the use of the BSPM technique as a medical diagnostic procedure. Furthermore, various types of TWA sources yielded distinct BSPM patterns of TWA amplitude. Therefore, it may be supposed that TWA mapping and pattern recognition could be used to help approximate regions within the heart that exhibit impaired repolarization.

Analysis of the susceptibility of clinically used ECG leads in TWA detection pointed to the V1 electrode location as the lead with the highest probability of TWA detection. However, *VMA* showed a higher ability for TWA detection when it was computed from all 12 lead ECG. Nevertheless, for the five regional sources of TWA located in the left ventricle, neither *VAA* nor *VMA* were indicative, even upon use of the BSPM technique, as AP duration changes in these regions were very slightly reflected on the thorax in comparison to other cases. We further showed that QT alternans could potentially develop via TWA generation processes, and that the highest influence on QT alternans arose from changes in mid-myocardial layers, potentially because these layers comprise the majority of the ventricular volume. In addition, the simulated discordant and regional AP duration changes in myocardial layers had a greater influence on TWA amplitude than the concordant TWA.

The reliable detection of TWA, as well as a better understanding of underlying process, may help in the identification of patients that are at high-risk of ventricular arrhythmia. This is particularly important in view of the relatively low positive predictive value of the TWA test, although the negative predictive value of the TWA test is very high. Indeed, the low positive predictive value of this test (19%) does not justify taking or omitting medical action [53]. In particular, this scenario applies to those patients qualified for protection by an ICD for the primary prevention of SCD. Obtaining a negative TWA test result may lead to a delay in the ICD implantation procedure by 1–2 years (until the next TWA survey). Thus, an improved TWA test, for example via the application of the two novel TWA-based parameters described in this paper, should increase this positive predictive value and would therefore be helpful in making decisions regarding the urgency of a given patient’s need for ICD implantation, according to the presence or absence of a presumably malignant form of TWA.

## Abbreviations

AI: alternans indicator
AP: action potential
APD: action potential duration
BSPM: body surface potential map
CAD: coronary artery disease,
CONC: concordant T-wave alternans
D_A/B: discordant T-wave alternans simulated in the apex and base
D_L/R: discordant T-wave alternans simulated in the left ventricle and right ventricle
D_P/A: discordant T-wave alternans simulated in the posterior and anterior wall
DISC: discordant T-wave alternans
ECG: electrocardiography
ICD: implantable cardioverter defibrillator
LVEF: left ventricle ejection fraction,
MI: myocardial infarction,
R_ANT: regional T-wave alternans simulated in the right basal anterolateral region (anterior)
R_INF: regional T-wave alternans simulated in the left mid inferior region (inferior)
R_LAN: regional T-wave alternans simulated in the left apical anterior region (left anterior)
R_LEF: regional T-wave alternans simulated in the left mid anterolateral region (left)
R_LLA: regional T-wave alternans simulated in the left mid lateral region (left lateral)
R_POS: regional T-wave alternans simulated in the left basal inferolateral region (posterior)
R_RAN: regional T-wave alternans simulated in the right mid anterolateral region (right anterior)
R_RIG: regional T-wave alternans simulated in the right basal lateral region (right)
R_SEP: regional T-wave alternans simulated in the right basal inferoseptal region (septum)
R_SUP: regional T-wave alternans simulated in the left mid anterior region (superior)
TWA: T-wave alternans
VAA: vector Angle Alternans
VF: ventricular fibrillation
VMA: vector Magnitude Alternans
VT: ventricular tachycardia
VVI: type of heart pacing: the ventricles are paced when the intrinsic ventricular rhythm falls below the pacemaker threshold
